# Projected Impact of a Sodium Consumption Reduction Initiative in Argentina: An Analysis from the CVD Policy Model – Argentina

**DOI:** 10.1371/journal.pone.0073824

**Published:** 2013-09-09

**Authors:** Jonatan Konfino, Tekeshe A. Mekonnen, Pamela G. Coxson, Daniel Ferrante, Kirsten Bibbins-Domingo

**Affiliations:** 1 Department of Health, Centro de Estudios de Estado y Sociedad, Buenos Aires, Argentina; 2 Directorate of Health Promotion and Control of Non Communicable Diseases, Ministry of Health, Buenos Aires, Argentina; 3 Department of Medicine, University of California San Francisco, San Francisco, California, United States of America; 4 University of California San Francisco Center for Vulnerable Populations at San Francisco General Hospital, University of California San Francisco, San Francisco, California, United States of America; 5 Department of Epidemiology and Biostatistics, University of California San Francisco, San Francisco, California, United States of America; Universidad Peruana de Ciencias Aplicadas (UPC), Peru

## Abstract

**Background:**

Cardiovascular disease (CVD) is the leading cause of death in adults in Argentina. Sodium reduction policies targeting processed foods were implemented in 2011 in Argentina, but the impact has not been evaluated. The aims of this study are to use Argentina-specific data on sodium excretion and project the impact of Argentina’s sodium reduction policies under two scenarios - the 2-year intervention currently being undertaken or a more persistent 10 year sodium reduction strategy.

**Methods:**

We used Argentina-specific data on sodium excretion by sex and projected the impact of the current strategy on sodium consumption and blood pressure decrease. We assessed the projected impact of sodium reduction policies on CVD using the Cardiovascular Disease (CVD) Policy Model, adapted to Argentina, modeling two alternative policy scenarios over the next decade.

**Results:**

Our study finds that the initiative to reduce sodium consumption currently in place in Argentina will have substantial impact on CVD over the next 10 years. Under the current proposed policy of 2-year sodium reduction, the mean sodium consumption is projected to decrease by 319–387 mg/day. This decrease is expected to translate into an absolute reduction of systolic blood pressure from 0.93 mmHg to 1.81 mmHg. This would avert about 19,000 all-cause mortality, 13,000 total myocardial infarctions, and 10,000 total strokes over the next decade. A more persistent sodium reduction strategy would yield even greater CVD benefits.

**Conclusion:**

The impact of the Argentinean initiative would be effective in substantially reducing mortality and morbidity from CVD. This paper provides evidence-based support to continue implementing strategies to reduce sodium consumption at a population level.

## Introduction

Cardiovascular diseases (CVD) are among the leading causes of death worldwide. [1] In Argentina, CVD is the number one cause of death in adults, accounting for nearly one-third of all deaths. [2] Population based strategies to reduce consumption of dietary sodium have been proposed to decrease the burden of these diseases and are considered by the World Health Organization (WHO) as a “best buy” for prevention of chronic conditions. However, few countries have developed, implemented, and evaluated CVD primary prevention policies and programs that include these dietary strategies. [3] Although no studies have measured sodium consumption in Argentina, consensus among experts suggests that current sodium intake is at least double of the WHO 2000 mg/day recommendation. [3] In addition to the sodium already added to processed and prepared food that account for a considerable portion of sodium intake in many countries, more than 25% of the population in Argentina add salt while eating. [4].

In 2011, a study by Ferrante et. al. demonstrated the feasibility of reducing sodium content in processed food in Argentina, [5] which supported the Ministry of Health (MoH) initiatives on sodium consumption. In 2011, large food companies have signed voluntary agreements with MoH to lower sodium content in processed foods, [6] the main source of sodium intake in Argentina. [7] The initiative called *Menos Sal Más Vida* (“Less Salt More Life”) requires food companies to reduce 5% to 15% of the sodium content of four groups of food: 1) processed meats; 2) cheese and dairy products; 3) soups and dressings; and 4) cereals, cookies, pizza and pasta (farinaceous). Although this campaign aims to reduce sodium intake in a two-year’s time frame, the impact has not been assessed to date. Recently, the first, locally representative sodium urinary excretion data in Argentina was released, providing a unique opportunity to assess daily sodium intake projecting the impact of the population based strategy to reduce sodium consumption. [8].

The aim of this study is to assess the projected impact of the current sodium reduction initiative being implemented in Argentina on CVD events and deaths and to evaluate the expected impact of this strategy from 2013 to 2023, as well as the additional projected impact of a continued sodium reduction approach over this entire decade.

## Methods

We used Argentina-specific data on 24-hour urinary sodium excretion, by sex [9], The La Pampa Pilot Study was an experimental study implemented in the Province of La Pampa, Argentina by the MoH that aimed to assess the impact of the voluntary sodium reduction of the restaurants and bakeries in the food they prepare. [8] We had access to the recorded urinary sodium excretion of a representative sample of 760 people at baseline in 2012 (before the intervention of the study), using a one-time spot urine sodium, a method that is correlated with 24 hour urinary sodium excretion. [10], [11] From the mean spot urine sodium concentrations (mEq/L) by gender, using La Pampa population-based data of age, weight, height and one-time spot urine creatinine we estimated the 24-hour urinary sodium excretion using a validated method for general population. [12] We assumed that daily excretion as the mean daily sodium consumption, since the high correlation between both measurements reported in the literature. [13], [14]. Although sodium consumption is known to vary by age, previous data from Argentina suggested that there were small differences between age groups [9]. Hence we estimated 24-hour urinary sodium excretion only by gender assuming the same age distribution.

To estimate the impact of the intervention, we used the CVD Policy Model-Argentina. The CVD Policy Model is a national scale, state-transition (Markov) computer simulation model of the coronary heart disease and stroke incidence, prevalence, mortality and costs in US adults 35–84 years old and was described elsewhere. [15] The model has been used to describe the trends in CHD and stroke and the effectiveness and cost savings gained from population-wide policies aimed at tobacco control, [16] and dietary salt. [17] The CVD Policy Model has been successfully adapted to create national CVD models for China [18] Argentina, [19]–[21] and recently, Mexico.

The model has three submodels: demographic–epidemiological, bridge and disease-history. The demographic–epidemiological submodel predicts the incidence of CVD and the rates of death due to causes other than CVD among persons without a history of CVD. CVD events are predicted based on age, sex and the following six factors: smoking status (active smoker or secondhand smoke exposed), systolic blood pressure (SBP), use of medication to lower blood pressure, level of high density lipoprotein (HDL) cholesterol, level of low density lipoprotein (LDL) cholesterol and presence or absence of diabetes. For persons in whom CVD develops, the bridge submodel characterizes the initial CVD event and related events for 30 days. The disease-history submodel then predicts the rate of subsequent CVD events and death rates from CVD and deaths not related to CVD among simulated subjects with CVD, with each category stratified according to age, sex and history of events. Table 1 summarizes the data sources for the CVD Policy Model-Argentina.

**Table 1:** Data sources for CVD policy model-Argentina.

Data	Source
Population of Argentina and incoming 35-year-old persons, 2010–2050	Argentina National Statistics and Census Institute [32]
**Incidence**	
Acute Myocardial Infarction (MI)	Population-based MI registry in a Buenos Aires district [33]
Stroke	Hospital admisión registry [2]
MI and stroke prevalence 2010	Population-based risk factor telephone survey in Buenos Aires [34]
**Mortality**	
Coronary Heart Disease*	Statistics and Information Department, Ministry of Health [2]
Stroke**	Statistics and Information Department, Ministry of Health [2]
**One-day and 28-day CHD case-fatality**	
CHD	Argentine national hospital survey [35] Ministry of Health admissions database [2]
Stroke	Argentine National Registry (RENACER) [36]
Stroke 28-day case fatality	Iquique Stroke Study (PISCIS) [37]
**Risk Factors**	
Means of Systolic Blood Pressure, LDL cholesterol and HDL cholesterol	CARMELA Study [38]
Smoking prevalence	2009 Second National Risk Factor Survey (Encuesta Nacional de Factores de Riesgo) [4]
Use of anti-hypertensive medicines	2009 Second National Risk Factor Survey (Encuesta Nacional de Factores de Riesgo) [4]

* International Classification of Diseases, 10th revision (ICD-10) codes: 10 I21, I22 Myocardial Infarction;, ICD-10 I20, I23–I25 angina and other CHD; I472, I490, I46, I50, I514, I515, I519, I709 of poorly defined cardiovascular disease events and death.;

** ICD-10 Codes I60–I69 for stroke deaths.

### Calibration of the CVD Policy Model-Argentina

The CVD Policy Model-Argentina was used to project the number of deaths was compared with actual CVD deaths observed from Argentina Vital Statistics for the years 1997–2009. We included definite CVD deaths plus a percentage of poorly defined deaths (named “garbage” codes) that could be attributed to CVD deaths. [22] We found a difference of less than 5% between the last total CVD deaths available from national statistics, compared with the first estimation of total CVD deaths from the CVD Policy Model. A detailed explanation about the model calibration process has been published elsewhere. [20].

#### Cardiovascular disease event prediction

For the main simulations, multivariate risk equations were estimated from US Framingham Heart Study data [23] with coronary heart disease (CHD) (including stable or unstable angina, non-fatal MI, fatal MI or arrest) or stroke events (ischemic stroke, including transient ischemic attack, plus hemorrhagic stroke) [24] as the outcome. Risk coefficients for age, sex, SBP, smoking status, LDL, HDL, diabetes and use of anti-hypertensive medicines were estimated in the CHD and stroke prediction model and age, sex, SBP, smoking status and diabetes in the total stroke model. [24] Statistically significant (p<0.05) age-by-risk-factor interactions were incorporated into age-specific risk factor coefficients. Risk factor beta coefficients were estimated from examinations 9 to 13, 24 and 25 from the original Framingham Heart Study cohort and 1–6 from the Framingham offspring cohort, including participants for whom adequate data were available for a time-dependent logistic regression analysis. Annual risk for CVD is calculated for each model cell by a multivariate logistic regression equation.

### Intervention

Since the “Less salt More Life” initiative aims to reduce 5–15% of sodium content in processed food, we used the mean value of that reduction (10%), applied to the 80% of sodium consumed that comes from processed foods. [7] We simulated two scenarios for ten years, 2013–2023:

1. Scenario 1 estimated the impact that the current initiative could have by reducing 8% of sodium consumption, 4% in the first two years (the timeframe that was agreed with the food industries) and then continued the projection without further interventions.
2. Scenario 2 estimated the impact this initiative could have if maintained for the 10 years, progressively reducing sodium consumption by 40%, 4% each year until 2022.

We assumed that each gram of reduction in salt consumption would decrease 1.87 mmHg in systolic blood pressure of hypertensive and older than 65 years old and 1.17 mmHg in systolic blood pressure of non-hypertensive and/or less than 65 years. [25].

## Results

### Sodium Consumption in Argentina

Using the spot urines from the Argentina La Pampa Pilot study, after estimating 24-hour urinary sodium excretion, we estimated a mean sodium consumption of 4832 mg/day for men, 3983 mg/day for women and 4407 mg/day on average, what is equivalent to 12.1, 10 and 11 grams/day of salt respectively. The derived population distribution of sodium consumption is shown in Table 2.

**Table 2:** Mean sodium consumption by age groups and sex and mean reduction of systolic blood pressure reducing 8% the sodium consumption (Scenario 1) and 40% the sodium consumption (Scenario 2).

sex	mean mg/day sodium consumption	sodium reduction (mg/day)	mean SBP (mmHg) reduction attributed to sodium reduction	
			young/non-HTN	HTN/elderly
scenario 1 (8% reduction)				
male	4832	387	1.13	1.81
female	3983	319	0.93	1.49
scenario 2 (40% reduction)				
male	4832	1933	5.65	9.04
female	3983	1593	4.66	7.45

SBP: Systolic blood pressure.

HTN: Hypertensive patients.

* : Less than 65 years.

** : 65 years and above.

n/a: Not applicable.

### Projected Impact of Sodium Reduction Policies on Sodium Intake and Blood Pressure

The initiative to reduce sodium consumption currently in place in Argentina is projected to have a substantial impact on sodium consumption and blood pressure from 2013–2023. The current initiative is projected to reduce the mean sodium consumption by 387 mg/day in men and 319 mg/day in women (Scenario 1). If this strategy was maintained for 10 years (Scenario 2) it is projected to reduce sodium intake by 1933 mg/day in men and 1593 mg/day in women. In terms of blood pressure reduction, we find that each gram of sodium reduction will have bigger effects in persons 65 years of age and older and hypertensives. Scenario 1 would reduce systolic blood pressure by 0.93 mmHg up to 1.81 mmHg depending on the population subgroup and Scenario 2 would reduce systolic blood pressure by 4.66 mmHg up to 9.04 mmHg depending on the subgroup. A more detailed description, by age groups and sex, is described in Table 2.

### Projected Impact

We estimated that the impact the current salt initiative implemented in Argentina (Scenario 1) would avert about 19,000 deaths of which 6,000 are CHD deaths and 2,000 are stroke deaths. We also projected that the “Less salt More Life” initiative can avert about 13,000 total myocardial infarctions and 10,000 total stroke cases from 2013–2023 (Table 3). These results represent a reduction of 0.6% in total mortality in 35 years and older adults, 1.5% in total myocardial infarctions and 1% in total stroke cases in the next decade.

**Table 3:** Absolute number of baseline and avoided events in Scenario 1 and 2 from 2013–2023.

	Total Deaths		CHD Deaths		MI		Stroke	
	Baseline	Avoided Events	Baseline	Avoided Events	Baseline	Avoided Events	Baseline	Avoided Events
Scenario 1*								
Men	1,555,000	−12,000	318,000	−4,500	488,000	−8,000	515,000	−5,500
								
Women	1,550,000	−7,000	275,000	−1,500	376,000	−5,000	495,000	−4,500
								
Total	3,100,000	−19,000	593,000	−6,000	864,000	−13,000	1,010,000	−10,000
Scenario 2‡								
Men	1,555,000	−35,000	318,000	−11,500	488,000	−25,500	515,000	−15,000
								
Women	1,550,000	−20,000	275,000	−4,500	376,000	−12,500	495,000	−12,000
								
Total	3,100,000	−55,000	593,000	−16,000	864,000	−38,000	1,010,000	−27,000

* Scenario 1 estimated the impact that the current initiative could have by reducing 8% of sodium consumption, 4% in the first two years (the timeframe that was agreed with the food industries) and then continued the projection without further interventions until 2022.

‡ Scenario 2 estimated the impact this initiative could have if maintained for the 10 years, progressively reducing sodium consumption by 40%, 4% each year until 2022.

In addition, if this sodium reduction strategy were continued for 10 years, setting progressive goals simulated in Scenario 2, the impact would be even greater: 55,000 deaths, with 16,000 CHD deaths and 5,000 stroke deaths, 38,000 total myocardial infarctions and 27,000 total strokes would be avoided in the next 10 years (Table 3). These reductions would translate into a decrease of 2% in overall mortality in 35 years and older adults, as well as a 4.3% in total myocardial infarctions and a 2.7% decrease in total strokes.

## Discussion

The impact of the Argentinean initiative “Less Salt More Life” to reduce sodium consumption would be significant as it would substantially reduce CVD related mortality and morbidity in Argentina. Moreover, there would be a much larger benefit in the reduction of CVD if this initiative was maintained progressively over 10 years, requiring industries to reduce, for instance, 5% of sodium in processed foods every year over this period. According to these results, the current initiative would decrease 0.4 grams (1 gram of salt) from the daily sodium consumption in Argentina and if a progressive sodium reduction strategy is maintained in Argentina for 10 years, a 1.8 grams of sodium (4.5 grams of salt) daily sodium consumption would be lowered, which would bring Argentina close to the WHO recommended consumption of 2000 mg/day of sodium (5 grams of salt a day). [3].

Previous modeling studies have demonstrated the effectiveness of sodium reduction strategies in the. [17], [26], [27] Using the CHD Policy Model in the US population, a 3 g/day reduction in dietary salt in the US is projected to avoided new cases of CHD by 60,000 to 120,000, stroke by 32,000 to 66,000 and could save $10–$24billioin in health care costs annually. This study found that an even greater benefit would be observed in blacks, women, and older adults. [17] More recently, another study projected the impact of sodium reduction on CVD and mortality in the US. This study found that population wide sodium reduction simulated in multiple scenarios is projected to avoid 280,000 to 500,000 CVD deaths and add 919 to 5970 years-of-life by over ten years. [26] Ferrante et al. demonstrated the cost-effectiveness of a population- based intervention to reduce sodium consumption in Argentina. [21] Although that analysis reported a bigger impact for avoided events than reported here, it projected a desirable 3 grams of salt reduction scenario and did not incorporate a critical variable in assessing potential benefit – baseline sodium consumption as measured by Argentina specific data on urinary sodium excretion. Except for these differences, the effects published of the salt intervention initiative in Argentina are consistent with our findings. We extend and add to this prior work by using an estimate for baseline sodium consumption in Argentina based on measured urinary sodium and modeling the actual Argentina policy initiative using the CVD Policy. Our work thus provides the best available estimates for the impact of the current policies underway in Argentina.

Some limitations of our study should be considered. We assumed the same sodium consumption in adults over 35 years accounting small differences reported in the literature for Argentina, [9] besides the differences in consumption could be observed. Additionally, we assumed that the sodium urine excretion representative of the Province of La Pampa reflected the sodium urine excretion of the entire Argentina, because of the lack of national representative data on urinary sodium excretion. Although there are no data about regional differences, it is possible that some small differences in sodium consumption could be found. Additionally, we should consider that some variations in population’s ‘sodium consumption and possible replacements by other product in response to the lower sodium content may occur over time that could influence final estimated impact of the intervention. While these estimation methods have their limitations, they allow us to consider baseline sodium consumption and the impact of the sodium reduction policies on the distribution of consumption, which is often lacking in other analyses. Finally, all modeling studies are limited by the integrity of their inputs. We used the best available data from Argentina (described in Table 1) to adapt the US CVD Policy Model to the Argentina context. We use data from Framingham to determine the association of CVD risk factors and CVD outcomes, rather than studies in a Latin American population. Prior studies adapting Framingham to other international contexts have suggested that the associations between risk factors and CVD events (beta ) appear to be the same in other populations, although actual event rates may differ (alpha) across populations [28] Event rates for the CVD Policy Model-Argentina have been calibrated with the best available epidemiological data from Argentina.

Some uncertainty about the optimal daily sodium intake remains and others [29], [30] have recently suggested the possibility of developing adverse consequences if sodium consumption is too low. Our data and projections suggest that the sodium consumption in Argentina is extremely high and certainly in the range that sodium reduction efforts are likely to yield cardiovascular benefit based on the multiple trials showing the benefits of sodium reduction on blood pressure. Although more primary data collection is necessary to determine if there are in fact adverse events associated with very low sodium consumption, the high starting average consumption in Argentina makes it extremely unlikely that these hypothesized adverse outcomes will have any effect on populations in Argentina. On the contrary, our projections suggest that the current voluntary agreements to decrease sodium content in processed food in Argentina could have a substantial impact on population health. Although the effect of this type of intervention may vary between countries, Argentina’s experience with public-private partnership for salt reduction could be successfully extended to other countries in the Pacific Rim and South American region. But, despite the effectiveness of this partnership to achieve processed foods with less sodium, governments should also consider public regulation and market intervention as important mechanisms to prevent diet-related chronic diseases. [31] Our projected health benefits assume that the initial sodium reductions achieved are maintained over the decade (in Scenario 1). However there remains considerable concern that the food industry may be reluctant to sustain even the initial sodium reduction after meeting the MoH obligations. To assure continued compliance, policy makers may consider levying a sanction to limit the sodium content in processed foods in Argentina, a regulatory policy that could at least assure that the initial reductions are maintained and could further be used to potential achieve larger population health benefits from continued sodium reductions as we model in Scenario 2.

This paper has the strength of projecting the potential health benefits of reducing sodium on a population wide level. In addition, our work generates evidence of the impact of a concrete health promotion, population-based intervention and provides evidence-based support to health policy-makers and government authorities to strengthen initiatives in place and continue to implement strategies to reduce sodium consumption in a progressive fashion. The development and dissemination of the Argentina experience may be a valuable tool to support the implementation of health promotion strategies in the region.
